# Effects of Ketamine on Learning and Memory in the Hippocampus of Rats through ERK, CREB, and Arc

**DOI:** 10.3390/brainsci11010027

**Published:** 2020-12-29

**Authors:** Mingxian Shi, Jiafeng Ding, Lin Li, Hui Bai, Xinran Li, Ling Lan, Honggang Fan, Li Gao

**Affiliations:** Heilongjiang Key Laboratory for Laboratory Animals and Comparative Medicine, College of Veterinary Medicine, Northeast Agricultural University, Harbin 150038, China; shimingxian2020@163.com (M.S.); jiafengding@cau.edu.cn (J.D.); w1980003197@163.com (L.L.); baihui101214@163.com (H.B.); lixinran@fosu.edu.cn (X.L.); ling139035213222@163.com (L.L.); fanhonggang2002@163.com (H.F.)

**Keywords:** ketamine, learning and memory, ERK, CREB, Arc

## Abstract

Ketamine has become a popular recreational drug due to its neuronal anesthesia effect and low price. The process of learning and memory is part of the distinctive high-level neural activities in animals. We investigated the effects of subanesthetic and anesthetic doses of ketamine on the learning and memory-related signal transduction mechanisms. We used the Morris water maze test to execute rats’ learning and memory ability and detected changes of Arc mRNA and Arc, cAMP-response element-binding protein (CREB), phospho-CREB (p-CREB), extracellular signal-regulated kinase (ERK), and phospho-ERK (p-ERK) protein expression in the hippocampus 10 min and 24 h after administration. Ten min after ketamine injection, the Arc gene and the protein expression levels increased in all groups; p-ERK only increased in the chronic subanesthetic dose group. After 24 h, the Arc gene and the protein expression levels of the subanesthetic dose group increased, but those of the chronic subanesthetic dose group and anesthetic dose group decreased. However, p-ERK increased in all groups. A chronic subanesthetic dose of ketamine could increase learning and memory ability through ERK, CREB, and Arc in a short time, and the high body temperature after the subanesthetic dose of ketamine injection was the main factor leading to changes in Arc. The subanesthetic dose of ketamine regulated learning and memory through ERK, CREB, and ARC 24 h after injection.

## 1. Introduction

Ketamine is an intravenous general anesthetic. It is clinically used as an anesthetic for surgery or anesthesia inducer. It has a certain mental dependence and impact on learning and memory [1,2]. Ketamine has an anesthetic effect at high doses (>80 mg/kg), but the drug can cause DNA damage in rodent models within 12 h [3]. At low doses, it can simulate an analgesic effect. Therefore, ketamine can be used as an analgesic after surgery [4]. Considering that ketamine has hallucinogenic and addictive properties, it often appears in various entertainment venues and has become a substance that is increasingly abused [5,6].

Learning and memory are important and basic functions of the brain. Scientists generally support that the hippocampus is an important brain area that plays a role in the formation of learning and memory [7]. It may play a role in the transition from short-term memory to long-term memory. In the nervous system, a large number of neurons are connected to one another between synapses to form neural circuits, which trigger a series of biochemical cascade reactions and lead to changes in synaptic plasticity, affecting learning and memory. The memory classification method can be divided into short-term memory and long-term memory on the basis of memory duration. Direct early genes (IEGs) are a class of proto-oncogenes that can encode transcription factors. They have the function of coupling short-term signals with long-term changes. They primarily include c-fos, c-jun, c-my, egr family, and Arc [8]. Among the IEGs, the cytoskeleton protein Arc/Arg3.1 plays an important role in the synaptic plasticity, learning, and memory in the mammalian brain. The dynamic regulation of Arc mRNA, including its destruction, modulates hippocampal behavior, GLUR1 signaling, and spine density [9,10,11,12,13,14].

Mitogen-activated protein kinase (MAPK) is a threonine/serine protein kinase in biological cells. It is an important medium for transmitting extracellular signals from the cell surface to the inside of the cell. Scientists have discovered four MAPK signal transduction pathways in eukaryotic cells. Among the pathways, the extracellular signal-regulated kinase (ERK) pathway plays an important role in the formation of long-term memory and learning memory in the brain [15,16]. ERK can activate cAMP-response element-binding protein (CREB) by activating ribosomal S6 kinase, thereby initiating CREB-dependent transcription. In the cytoplasm and dendrites, ERK can regulate transcription factors, thereby indirectly inducing protein synthesis [17]. Its activation leads to changes in the morphology of hippocampal dendrites. Therefore, ERK may be involved in the formation of neuronal plasticity, which promotes the reception and transmission of information.

To date, no comparative study has been conducted on the effect of the subanesthetic dose and anesthetic dose of addictive anesthetic ketamine on learning and memory-related signal transduction mechanism. Therefore, we investigate the effect of ketamine on learning and memory in rats and its mechanism from the perspectives of subanesthetic dose, chronic subanesthetic dose, and anesthetic dose 10 min and 24 h after ketamine injection. Our research provides new insights into the mechanism of ketamine’s influence on learning and memory and helps to prompt the clinical applications of ketamine.

## 2. Materials and Methods

### 2.1. Drugs

Ketamine was provided by the Department of Veterinary Surgery, Northeast Agricultural University.

### 2.2. Animals

Fifty-four young male Wistar rats, weighing 220–240 g, were purchased from the Laboratory Animal Center of Harbin Medical University. All the rats were given proper water and housed in an environment with temperature of 22 °C ± 1 °C, relative humidity of 50% ± 1%, and a light/dark cycle of 12/12 h. All animal studies (including the rat euthanasia procedure) were performed in compliance with the regulations and guidelines of Northeast Agricultural University Institutional Animal Care and conducted according to the Association for Assessment and Accreditation of Laboratory Animal Care and Institutional Animal Care and Use Committee guidelines.

### 2.3. Drug Administration

A total of 54 Wistar rats were acclimatized to the environment for 1 week. Subsequently, the rats were randomly divided into nine experimental groups (n = 6/group). In order to explore the effect of ketamine administration for 10 min, we set up 5 groups: control group (CG), subanesthetic dose group (K20G), anesthesia dose group (K80G), chronic subanesthetic dose group (CK20G), and high-temperature group (HG). In order to explore the effects of ketamine administration 24 h later, we set up another 4 groups: control group (CG), subanesthetic dose group (K20G), anesthesia dose group (K80G) and chronic subanesthetic group (CK20G). The subanesthetic dose and anesthesia dose groups were intraperitoneally administered with 20 and 80 mg/kg ketamine. The CK20G was intraperitoneally administered with 20 mg/kg for 20 days. The HG was exposed to a high-temperature environment for 10 min to increase its body temperature to simulate the phenomenon of increased body temperature after injection of a subanesthetic dose of ketamine. The CG was injected with a corresponding volume of normal saline. The ketamine was diluted with normal saline.

### 2.4. Morris Water Maze Test

In order to study the effects of ketamine on rats after 24 h, we set up four groups (CG, K20G, CK20G, and K80G) and performed the Morris water maze test. Within 20 days of continuous administration in CK20G, the rats in CG, K20G, and K80G were not treated with drugs. From the 16th day to the 20th day, rats were trained in the standard Morris water maze with a hidden platform before the rats in CK20G were injected with drugs every day. The equipment consisted of a hidden white platform (10 cm in diameter and 1.5 cm below the water surface in the center of the SW quadrant). The Morris water maze consisted of a black circular pool filled with warm (23 °C–25 °C), opaque water. The area of the maze was divided into four equal-sized quadrants: NE, NW, SE, and SW. At D16–D20, each rat underwent four trial sessions per day (60–70 min inter-trial interval) for five consecutive days. Each rat was successively placed into the water of each of the four quadrants, and a maximum of 90 s was allowed to find the platform. If rats failed to find the platform within this time limit, then they were guided to the platform and kept in the platform for 10 s. All trials were videotaped. Furthermore, a video track program allowed us to measure the time required to find the platform and other behavioral information of the spatial memory test (Coulbourne Instruments; Allentown, PA, USA). Therefore, we could use the output data for statistical analysis. On the 20th day of feeding (the 5th day of training), rats were dried with an electric hair dryer after training and injected with corresponding dose of drugs. A probe trial was performed 24 h after the injection, where the platform was removed from the pool to assess memory retention for the location of the platform. During the 90 s test trial, we recorded and analyzed the swimming path tracks and the number of platform crossings [18].

### 2.5. Tissue Preparation

After the injection of drugs, the rats were euthanized at each corresponding time point. After the scissors extended through the occipital foramen, the occipital bone and rat’s nasal bone were cut. The scissors entered from the nasal cavity, cut to both eyes to stop, and then, we separated the scissors to the sides along the occipital bone and parietal bone from the midline. Tweezers was used to open the parietal bones on both sides to fully expose the complete brain. After careful removal, the surface blood was rinsed with 0.01 M phosphate buffered solution (PBS). The brain was placed on a previously frozen ice tray, and the cerebral cortex was peeled off with an ophthalmic scissors to expose and isolate the hippocampal tissue. The removed hippocampal tissues were placed in diethypyrocarbonate (DEPC)-treated cryopreservation tubes. The cryopreservation tube was rapidly cooled in liquid nitrogen and then stored at −80 °C.

### 2.6. Protein Immunoblot Detection

SDS-PAGE and Western blot analyses used the following primary antibodies. The reents were purchased as follows: Glyceraldehyde-3-phosphate dehydrogenase (GAPDH) (14C10) Rabbit mAb (cat. #2118, Cell Signaling, Beverly, MA, USA), Phospho-p44/42MAPK (Erk1/2) Rabbit mAb (cat. #4370, Cell Signaling, USA), p44/42 MAPK (Erk1/2) Rabbit mAb (cat. #4695, Cell Signaling, USA), CREB (48H2) Rabbit mAb (cat. #9197, Cell Signaling, USA), Purified Mouse Anti-Arc (cat. #612602, San Jose, CA, USA), and Rabbit monoclonal [E113] to CREB (phospho S133; cat. #ab32096, Abcam, Cambridge, MA, USA).

The results were scanned into the computer, and we used Image J software to analyze the gray value of the protein strip.

### 2.7. RNA Extraction and Real-Time Quantitative Polymerase Chain Reaction (RT-PCR) Analysis

The mRNA expression of Arc was detected by quantitative RT-PCR. The PCR primers are shown in Table 1. GAPDH mRNA expression was used as an internal control to quantify the relative mRNA expression of target genes.

### 2.8. Statistical Methods

Statistical analysis and the generation of graphs were carried out using GraphPad Prism 5 software (San Diego, CA, USA). Statistical analysis was performed using one-way ANOVA. There were two time points in this study, 10 min and 24 h. Different treatment groups were compared with the control group within a time point. All data were presented as mean ± standard deviation (SD). In this study, *p* < 0.05 was considered significant.

## 3. Results

### 3.1. Effects of Ketamine on the Behavior of Rats

**Figure 1:**
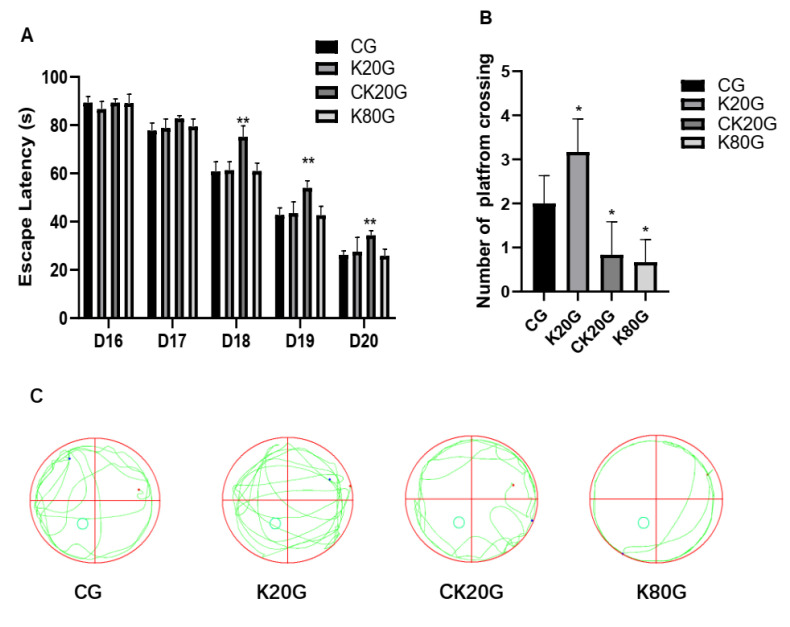
Effect of different doses of ketamine on the behavior of rats. (**A**) Escape latency from D16 to D20. Different treatment groups are compared with the control group on each day. (**B**) The number of platform crossings at the 24th hour after ketamine injection. (**C**) Swimming path tracks at the 24th hour after ketamine injection. Data are expressed as means ± SD. * *p* < 0.05 and ** *p* < 0.01 versus CG.

We conducted the Morris water maze test to evaluate the learning and memory ability of rats from the perspective of behavior. The effects of ketamine exposure on spatial learning and memory in rats are shown in Figure 1 and Table 2. The Morris water maze test data (Figure 1A) showed no significant differences in escape latencies in different groups in D16 and D17. However, from D18 to D20, escape latencies of the CK20G were significantly longer compared with that of the CG. In the spatial probe test data (Figure 1B,C), the number of platform crossings was lesser in the CK20G and K80G compared with that in the CG but more in the K20G.

### 3.2. Effect of Ketamine Administration on Arc Gene and Protein Expression in the Hippocampus

**Figure 2:**
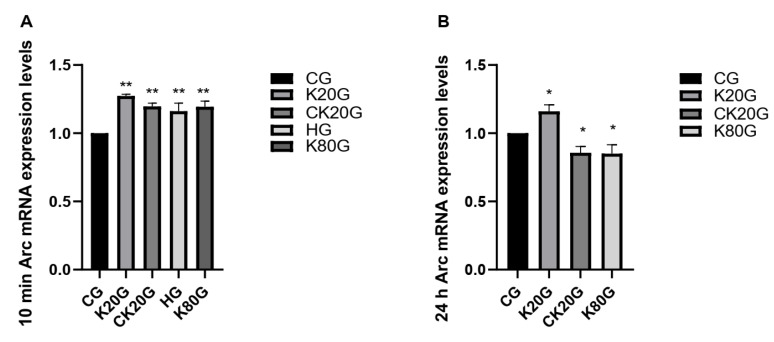
Effect of ketamine on Arc mRNA. (**A**) The Arc mRNA expression levels after 10 min. (**B**) The Arc mRNA expression levels after 24 h. Data are expressed as means ± SD. * *p* < 0.05 and ** *p* < 0.01 versus CG.

**Figure 3:**
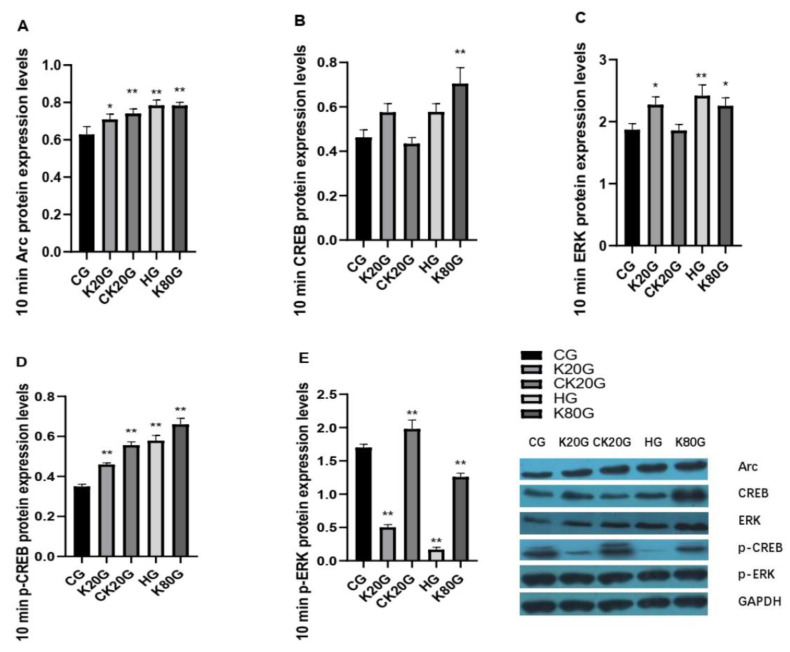
Expression levels of learning and memory-related proteins 10 min after ketamine injection. (**A**) The Arc protein expression levels after 10 min. (**B**) The cAMP-response element-binding protein (CREB) protein expression levels after 10 min. (**C**) The extracellular signal-regulated kinase (ERK) protein expression levels after 10 min. (**D**) The p-CREB protein expression levels after 10 min. (**E**) The p-ERK protein expression levels after 10 min. Data are expressed as means ± SD. * *p* < 0.05 and ** *p* < 0.01 versus CG.

**Figure 4:**
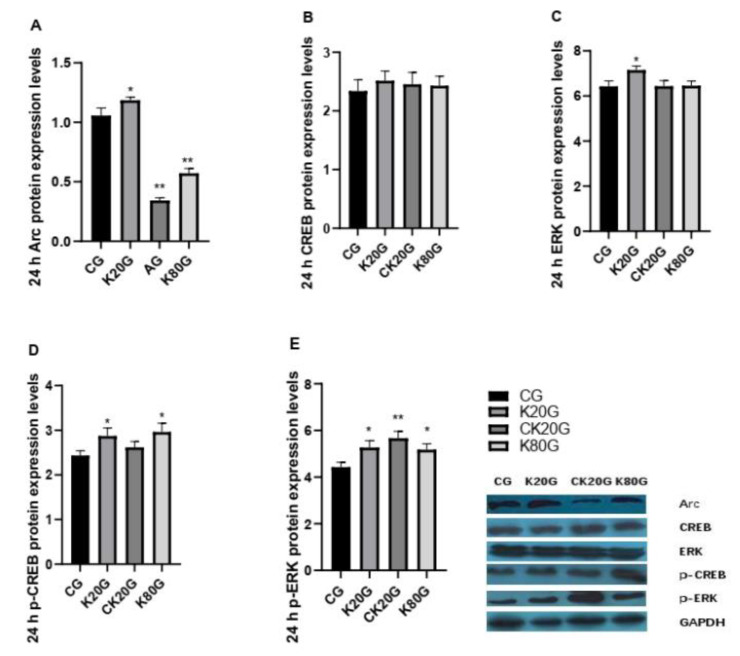
Expression levels of learning and memory-related proteins 24 h after ketamine injection. (**A**) The Arc protein expression levels after 24 h. (**B**) The CREB protein expression levels after 24 h. (**C**) The ERK protein expression levels after 24 h. (**D**) The p-CREB protein expression levels after 24 h. (**E**) The p-ERK protein expression levels after 24 h. Data are expressed as means ± SD. * *p* < 0.05 and ** *p* < 0.01 versus CG.

Arc is an important learning and memory-related indicator. We tested the mRNA (Figure 2 and Table 3) and protein content of Arc in the hippocampus of rats (Figure 3A, Table 4, Figure 4A and Table 5). Consequently, the change trend of the Arc mRNA and protein content of each group in the hippocampus was consistent. After 10 min of ketamine administration, the Arc mRNA and protein expression levels in all groups were significantly increased compared with that in the CG.

After 24 h of ketamine administration, the Arc mRNA and protein expression levels in the hippocampus in K20G were significantly increased compared with that in CG, whereas the Arc mRNA and protein expression levels in CK20G and K80G were significantly decreased compared with that in CG. The expression results of Arc gene and protein coincided with the results of the Morris water maze test in rats.

### 3.3. Detection of ERK–CREB Pathway-Related Indexes after Ketamine Administration

We hypothesize that ERK–CREB might be involved in the formation of neuronal plasticity, thereby affecting the ability of learning and memory. Therefore, we tested ERK and CREB pathway-related proteins and found that after 10 min of ketamine administration, the level of CREB protein in the hippocampus in K80G was significantly higher than that in CG, and the difference was significant. Compared with CG, the phospho-CREB (p-CREB) protein in all groups was significantly increased. ERK protein in the hippocampus in K20G and K80G had significant differences compared with that in CG, and only HG had a significant difference. The phospho-ERK (p-ERK) protein in K20G, HG, and K80G was significantly lower than that in CG, whereas the p-ERK protein in CK20G was significantly higher than that in CG (Figure 3B–E and Table 4).

After 24 h of ketamine administration, no significant difference in CREB protein was found in the hippocampus. The p-CREB protein in the hippocampus in K20G and K80G showed a significant increase. Compared with CG, the ERK protein in the hippocampus was only increased in K20G with a significant difference, whereas no significant changes were observed in other groups. The variation trend of the p-ERK protein was higher in each group, and the differences were significant compared with CG (Figure 4B–E and Table 5).

## 4. Discussion

Ketamine, an N-methyl-D-aspartic acid receptor antagonist, is commonly used in many species for its sedative and analgesic properties [19]. It can be administered intramuscularly, intraperitoneally, or intravenously to relieve pain. Ketamine is not only an anesthetic, but also a popular abused drug [20,21]. In clinical applications, studies have shown that ketamine is also a potential and effective antidepressant [22,23]. After the injection of ketamine, animals can produce psychiatric and clinical symptoms, such as increased sexual impulse. Furthermore, these symptoms are dose-dependent [24].

In our study, through consulting the literature and pre-experiments, we determined 80 mg/kg as the anesthetic dose and 20 mg/kg as the subanesthetic dose. Based on comparison, after 20 mg/kg ketamine administration, the head swung back and forth, and the body temperature increased and reached the highest value at the 10th minute. Thus, we set the 10th minute and 24th hour after the administration as the time points of our study.

Trujillo’s study showed that multiple injections of ketamine led to increased movement and significantly increased sensitivity to behavior, particularly when the experimenters placed the rats in the new environment. When a high dose of ketamine was given, the rats exhibited behavioral ataxia, but after repeated injections over a period of time, the rats showed a degree of tolerance to ataxia. This tolerance and sensitization are a manifestation of the different stages of ketamine addiction [25].

Therefore, in this study, rats were injected with ketamine at a subanesthesia dose with 20mg/kg continuously for 20 days to study the effect on the learning and memory ability of rats. In addition, the anesthetic dose of ketamine was included in the study to evaluate the similarities and differences in the mechanism of action between the two doses.

Ketamine abuse primarily affects brain tissue, causing mental problems such as anxiety, confusion, and memory loss [26]. Arc/Arg3.1 fosters the maturation of hippocampal network activity necessary for learning and memory storage [27]. The destruction of Arc mRNA can affect the learning and memory function of hippocampus [12]. Some anesthetics have been reported to inhibit the transcription of a large number of genes, including the Arc, thereby affecting memory consolidation [28]. In addition, Penrod RD’s study confirmed that Arc might play a regulatory role in drug addiction [29]. Therefore, in this experiment, Arc can be used as an important indicator to evaluate the learning and memory ability of rats.

The results of the Morris water maze test at the 24th hour after the administration of ketamine showed that during the spatial cognition experiment, CK20G has shown low learning ability from D18, which might be due to the long-term subanesthetic dose injection of ketamine. The brain’s learning function is impaired. In the spatial probe test, CK20G and K80G showed a decline in memory ability, but the memory ability of K20G rats was significantly higher than that of CG and still showed a clear state of excitement. The Arc level was consistent with the behavioral results of rats, which demonstrated the important role of Arc in learning and memory in rats.

Firstly, we reported the effects of a subanesthetic dose of ketamine and high body temperature on the learning and memory signaling pathway in rats. Robert A. Whittington illuminated the anesthetic induction of Arc in his paper through research of gene and protein expression and reduction mechanism, and he proved that isoflurane and propofol anesthesia reduce the Arc protein expression in the hippocampus of rats and mice. These changes were secondary to anesthesia-induced hypothermia phenomenon, thereby indicating that low temperature itself had a direct influence on Arc protein levels. This effect was due to the hypothermia inhibition of ERK/MAPK, resulting in decreased Arc mRNA expression [28].

In our study, we focused on rats’ head shaking and other vigorous movements when giving subanesthetic dose of ketamine, which were accompanied by an increase in body temperature. At the 10th minute, the body temperature stabilized at its peak. Thus, we placed the rats in a high-temperature environment to simulate the high body temperature. The results of the study showed that 10 min after ketamine administration, the changes in gene and protein expression in HG and K20G had the same trend compared with CG. p-ERK was significantly reduced, and p-CREB and Arc increased significantly because the high body temperature caused changes in the learning and memory-related indicators of rats. However, the change of p-ERK was negatively correlated with Arc. Twenty-four hours after ketamine injection, p-ERK was positively correlated with the changes of p-CREB and Arc. In Robert A. Whittington’s study, hypothermia induced by isoflurane and propofol anesthesia inhibited ERK activation and caused the low Arc expression in the hippocampus. From the experimental results, we thought that the change of Arc in the hippocampus of rats 10 min after the administration of ketamine was not caused by the activation of the ERK pathway. The high body temperature induced by the ketamine injection caused the change of Arc content in the hippocampus of rats. Twenty-four hours after the excitatory dose of ketamine was injected, the elevation of Arc was positively correlated with the ERK–CREB pathway. The difference between our experiment and Robert A. Whittington’s experiment was that Robert studied anesthesia. The anesthesia time was longer, and long-term hypothermia had a greater impact on rats. Our experiment focused on a subanesthetic dose experiment and the effect of drugs. The effect was maintained for a short time, and the body had a strong regulatory effect on short-term temperature stimulation. Therefore, our experiment did not set the 24 h detection time point for the high-body temperature group.

Secondly, we discussed the effects of the addictiveness of subanesthetic dose of ketamine on the learning and memory signaling pathway in rats. Considering that ketamine was addictive, we designed the chronic subanesthetic group. After continuous administration of a stimulant dose of ketamine for 20 days, we found that 10 min after administration, p-ERK, p-CREB, and Arc were positively correlated. However, 24 h after administration, the behavior and Arc content indicated that the long-term use of ketamine would cause a decline in learning and memory ability, but at this time point, p-ERK was in an activated state. Lv XF research indicated that Arc/Arg3.1 in the nucleus accumbens shell mediated the reconsolidation of morphine-associated context memory, and the local activation of the ERK–CREB signal pathway was the upstream mechanism of Arc/Arg3.1 [30]. This mechanism matched the 10th minute result. Notably, the sampling time point of rats in the Lv XF experiment was 30 min after administration. However, using the same method of administration and dose but a different sampling time point, the final mechanism was also different. Thus, we hypothesized that other proteins might join the formation and maintenance of memories in this 24th hour study, for example, cAMP-dependent protein kinase (PKA) [31,32]. Wimmer ME’s study showed that the cAMP–PKA–CREB signal pathway is also involved in memory [33]. Therefore, the mechanism of action of the drug might change because of the difference in time.

Finally, we analyzed the effect of an anesthetic dose of ketamine on the learning and memory signaling pathways in rats. Interestingly, 10 min after the administration of an anesthetic dose of ketamine, the expression of p-CREB and Arc increased, although the expression of p-ERK mRNA and protein decreased significantly. By contrast, after 24 h, p-ERK increased and Arc decreased. The result indicated that at the 10th minute of the initial administration, the drug showed a stimulating effect on the body, and the body showed a state of excitement. Compared with Robert A. Whittington’s experiment, our rats were investigated 24 h after the administration. The Arc content in the hippocampus and behavioral aspects of the rats did not return to the normal state after 24 h, which caused the body to show a state of reduced protein expression and decreased learning and memory ability. In Robert’s experiment, different drugs and anesthesia were used [28]. Inhaled anesthetics might be more easily metabolized by the body and the Arc expression returned to the normal level after 24 h, whereas the injection of ketamine caused a decline in Arc gene and protein expression, behavioral inhibition, and a decline in learning and memory abilities 24 h after administration, but this reduction was not caused by the ERK–CREB pathway.

## 5. Conclusions

We found that a chronic subanesthetic dose of ketamine could lead to a decline in learning and memory when ketamine was injected for 10 min. The regulation of learning and memory ability was through ERK, CREB, and Arc in CK20G. The change of body temperature might be the main factor of Arc change in the excitatory dose of ketamine. Twenty-four hours after ketamine injection, the ERK, CREB, and Arc were involved in regulating hippocampal learning and memory in K20G. After ketamine injection, the body responded to the drug in a short period of time, and the mechanism of action to the drug after 24 h was different. Therefore, in future scientific research, we should consider that when studying the mechanism of action of drugs on the body, the pathways corresponding to different time points might also change.

## Figures and Tables

**Table 1:** Primer sequence and amplification length of destination fragment.

Primer	Primer Sequence (5′ to 3′)	Products Size
GAPDH(F)	AGAACATCATCCCTGCATCC	101
GAPDH(R)	GGTAGGAACACAAAAGGCCA	
Arc(F)	ACTCCCTACTGTTGATCTGTTTGCTCC	109
Arc(R)	ACCCGTCATTTTCTCTGCCCTTTGA	

**Table 2:** Table for ANOVA results for behavior analysis data.

Behavior Analysis	F Value	Mean Square	*p* Value
D16	(3, 20) = 1.248	10.52	*p* = 0.3187
D17	(3, 20) = 2.420	21.50	*p* = 0.0962
D18	(3, 20) = 19.91	301.1	*p* < 0.0001
D19	(3, 20) = 3.929	98.84	*p* = 0.0244
D20	(3, 20) = 7.540	94.72	*p* = 0.0014
Number of platform crossing	(3, 20) = 18.02	8.111	*p* < 0.0001

**Table 3:** Table for ANOVA results for Arc mRNA data.

Arc mRNA	F Value	Mean Square	*p* Value
10 min	(4, 25) = 26.01	0.03049	*p* < 0.0001
24 h	(3, 20) = 29.68	0.06418	*p* = 0.0001

**Table 4:** Table for ANOVA results for expression levels of proteins 10 min after ketamine injection data.

Protein	F Value	Mean Square	*p* Value
ARC	(4, 25) = 14.46	0.01247	*p* = 0.0004
CREB	(4, 25) = 17.46	0.03490	*p* = 0.0002
ERK	(4, 25) = 11.61	0.1941	*p* = 0.0009
p-CREB	(4, 25) = 104.8	0.04276	*p* < 0.0001
p-ERK	(4, 25) = 343.1	1.787	*p* < 0.0001

**Table 5:** Table for ANOVA results for expression levels of proteins 24 h after ketamine injection data.

Protein	F Value	Mean Square	*p* Value
ARC	(3, 20) = 274.5	0.4749	*p* < 0.0001
CREB	(3, 20) = 0.4808	0.01575	*p* = 0.7046
ERK	(3, 20) = 7.951	0.3797	*p* = 0.0087
p-CREB	(3, 20) = 6.991	0.1731	*p* = 0.0126
p-ERK	(3, 20) = 11.51	0.8028	*p* = 0.0028

## Data Availability

All data included in this study are available upon request by contact with the corresponding author.
